# Using Structural Analysis *In Silico* to Assess the Impact of Missense Variants in MEN1

**DOI:** 10.1210/js.2019-00260

**Published:** 2019-09-27

**Authors:** Richard C Caswell, Martina M Owens, Adam C Gunning, Sian Ellard, Caroline F Wright

**Affiliations:** 1 Institute of Biomedical and Clinical Science, University of Exeter School of Medicine, Exeter, United Kingdom; 2 Department of Molecular Genetics, Royal Devon and Exeter NHS Foundation Trust, Exeter, United Kingdom

**Keywords:** multiple endocrine neoplasia type 1, protein structure, missense variant interpretation, genomics

## Abstract

Despite the rapid expansion in recent years of databases reporting either benign or pathogenic genetic variations, the interpretation of novel missense variants remains challenging, particularly for clinical or genetic testing laboratories where functional analysis is often unfeasible. Previous studies have shown that thermodynamic analysis of protein structure *in silico* can discriminate between groups of benign and pathogenic missense variants. However, although structures exist for many human disease‒associated proteins, such analysis remains largely unexploited in clinical laboratories. Here, we analyzed the predicted effect of 338 known missense variants on the structure of menin, the *MEN1* gene product. Results provided strong discrimination between pathogenic and benign variants, with a threshold of >4 kcal/mol for the predicted change in stability, providing a strong indicator of pathogenicity. Subsequent analysis of seven novel missense variants identified during clinical testing of patients with MEN1 showed that all seven were predicted to destabilize menin by >4 kcal/mol. We conclude that structural analysis provides a useful tool in understanding the effect of missense variants in *MEN1* and that integration of proteomic with genomic data could potentially contribute to the classification of novel variants in this disease.

The rapid expansion in recent years of genomic data from both patient and control groups has vastly improved the quantity and quality of information available to clinicians who are attempting to classify novel genetic variants. Although interpretation of likely loss-of-function variants such as stop-gain or frameshift variants is often straightforward, the same is not true of missense variants, for which the effect of an amino acid substitution is likely to be specific to its context in the protein of interest. Moreover, such variants are often rare or unique and thus must be interpreted on a case by case basis.

Numerous methods have been developed for predicting the phenotypic effect of missense variants. As has been comprehensively reviewed elsewhere [1], these methods rely on analysis of DNA and protein conservation, protein structure-based analysis, or a combination of the two. In the latter, widely used tools such as PolyPhen may incorporate information on the nature of the amino acid change itself (*e.g.,* Grantham distance between native and variant amino acids, changes in polarity or charge), effects on predicted secondary structure, and where available, data derived from the structural context, such as changes in hydrogen bonding or atomic crowding. However, such data are used in a qualitative, rule-based manner in the final prediction [1], and the tools most widely used in the clinical setting do not specifically address the quantitative effects of missense variants on protein stability. Nevertheless, these effects can be calculated when there is an experimental or modeled three-dimensional structure for the protein of interest; FoldX [2], Rosetta [3, 4], and other computational methods have been widely used by structural biologists to investigate the effects of missense variants on protein folding and stability [5, 6]. Despite this, few studies have addressed whether there is a direct clinical application of such an approach (*i.e.,* whether pathogenic and benign variants can be distinguished on the basis of their predicted effects on thermodynamic stability).

The potential utility of protein stability data in the analysis of missense variants was recently demonstrated in studies of the Lynch syndrome protein MSH2 [7] and in phenylalanine hydroxylase (PAH) [8], in which pathogenic variants result in phenylketonuria. Both of these studies combined *in silico* analysis with extensive functional analysis of a number of *MSH2* and *PAH* variants; however, resources for the latter are unlikely to be routinely available in clinical genetics laboratories. We therefore asked whether *in silico* analysis, based predominantly on the predicted effects of missense variants on protein stability, can help discriminate between pathogenic and benign variations in the context of clinical testing of the *MEN1* gene.

Pathogenic variants in the *MEN1* gene cause multiple endocrine neoplasia type 1 (MEN1), an autosomal dominant disorder in which patients develop neoplastic lesions in various endocrine tissues, principally the parathyroids, pituitary, and pancreas [9]. Pathogenic variants may be either inherited or acquired; in both cases, however, development of disease requires loss of heterozygosity consistent with a role for menin, the *MEN1* gene product, as a tumor suppressor. The biological activity of menin is not fully understood, but it is known to bind to and inhibit the activity of JunD [10], a component of the proliferation-associated transcription factor AP-1. Loss of menin activity is presumed to result in deregulated activity of AP-1, leading to increased cell proliferation and ultimately neoplasia. Menin also regulates gene expression via interaction with the histone methyltransferase KMT2A (MLL1) and forms an essential component of the MLL complex, which upregulates expression from target genes including those of the *HOX* cluster [11]. Menin may also play a role in DNA damage repair via an interaction with FANCD2, and loss of activity has been shown to result in increased sensitivity to DNA damage [12]. Finally, menin has been shown to repress telomerase activity, and depletion of menin in human fibroblasts results in their immortalization [13]. Thus, loss of menin activity could lead to neoplasia and tumor formation via a number of potential pathways.

The most common presenting feature in patients with MEN1 is hyperparathyroidism, which occurs in ∼95% of these patients as a result of tumors of the parathyroid gland; however, tumors are also frequently observed in the pancreatic islets (40% to 70% of patients) and pituitary (30% to 40% of patients) [14]. Patients may also develop tumors of the adrenal cortex, carcinoid tumors, and nonendocrine tumors, including lipomas, angiofibromas, collagenomas, and meningiomas [15], resulting in a range of clinical symptoms that may overlap with those of diseases of different genetic etiology [16–18]. This overlap presents one of the key problems in assessing genetic variants in cases of MEN1. Although a large number of pathogenic variants have been reported in *MEN1*, genetic testing continues to uncover novel missense substitutions that require assessment of their potential pathogenicity. A further confounding issue is the often later onset of disease, with reported age-related penetrance of 10% to 43% at 20 years and 81% to 94% by 50 years [14, 19], which may lead to apparent nonsegregation of a variant with disease within a family pedigree.

The identification of a genetic etiology has important implications for the patient and for his or her family members. With the exception of pituitary neuroendocrine tumors, MEN1-associated tumors are usually multiple; treatment is therefore challenging, requiring a multidisciplinary team of experts to reduce morbidity and mortality [20]. The identification of the familial disease-causing variant enables the identification of carriers when they are still asymptomatic. Clinical surveillance in these individuals allows early recognition of the clinical manifestations and therapeutic intervention. For example, primary hyperparathyroidism often remains asymptomatic in many patients, but prolonged hypercalcemia usually results in bone loss and/or nephrocalcinosis [21].

Approximately 20% of the variants identified in the *MEN1* gene are missense variants [22]. The standards and guidelines published by the American College of Medical Genetics and Genomics and the Association for Molecular Pathology describe a framework for the classification of sequence variants [23]. Adjustments to this framework have been proposed for the interpretation of *MEN1* missense variants [24]. However both agree that variants of uncertain significance should not be used to guide the clinical management of patients. This could lead to an underdiagnosis of MEN1 and a lost opportunity for screening of at-risk relatives. For these reasons, methods that assist in the classification of variants in *MEN1* are of clinical value. The availability of a number of experimental structures for menin, the *MEN1* gene product, raises the possibility that structural analysis may provide such clinical utility.

We report here that thermodynamic analysis of *MEN1* variants *in silico* provided a very strong positive predictive value (PPV) for pathogenicity, thereby helping to assess the effect of novel missense variants on protein function and potentially allowing the use of such analysis in variant classification. We also discuss briefly the wider application of this approach to other diseases.

## 1. Materials and Methods

### A. Variant Groups, Transcripts, and Numbering

Lists of previously reported inherited missense single nucleotide variants in *MEN1* were downloaded from the Human Gene Mutation Database, Professional version (HGMD Pro) [25]; the Genome Aggregation Database (gnomAD) [26], and the Sydney Genomics Collaborative Database (SGCD) [27]. For the purposes of this analysis, variants were divided into groups as follows: pathogenic: “disease mutation” (DM) class variants reported in the HGMD Pro but not in the gnomAD or SGCD (n = 162); benign: variants reported in the gnomAD or SGCD but not as DM class in the HGMD Pro (n = 206); and uncertain: variants reported as DM in the HGMD Pro and present in the gnomAD and/or SGCD (n = 14). Different nucleotide substitutions resulting in the same coding change were regarded as a single missense substitution. In addition to these previously reported variants, analysis was performed on seven novel missense variants: H46P, A164P, L175P, A345P, I360F, F364S, and G419D (see Table 1 for details). These variants were identified in our laboratory as part of the National Health Service (England) Genetic Testing Service for rare inherited diseases. The patients tested fulfilled the criteria for a clinical diagnosis of MEN1 [14], presenting with at least two of the three main MEN1-associated endocrine lesions or one typical MEN1-associated tumor and a first-degree relative with MEN1 or a MEN1-associated lesion at a young age. For patients with a family history, the relevant variants (H46P, A164P, I360F, and F364S) were all shown to cosegregate with disease in the family. Informed consent for genetic testing was obtained from all subjects.

**Table 1.** Details of Seven Novel Missense Variants in *MEN1*

Variant No.	1	2	3	4	5	6	7
HGVS c. notation	c.137A>C	c.490G>C	c.524T>C	c.1033G>C	c.1078A>T	c.1091T>C	c.1256G>A
HGVS p. notation	p.(His46Pro)	p.(Ala164Pro)	p.(Leu175Pro)	p.(Ala345Pro)	p.(Ile360Phe)	p.(Phe364Ser)	p.(Gly419Asp)
Genomic variant (GRCh37/hg19)	chr11:64577445T>G	chr11:64575527C>G	chr11:64575493A>G	chr11:64573720C>G	chr11:64573214T>A	chr11:64573201A>G	chr11:64572600C>T
Reported in gnomAD?	No	No	No	No	No	No	No
SIFT prediction	Damaging	Damaging	Damaging	Damaging	Damaging	Damaging	Damaging
PROVEAN prediction	Deleterious	Deleterious	Deleterious	Deleterious	Deleterious	Deleterious	Deleterious
PolyPhen prediction	Probably damaging	Probably damaging	Probably damaging	Probably damaging	Probably damaging	Probably damaging	Probably damaging
REVEL score	0.894	0.925	0.965	0.909	0.883	0.945	0.912

All variants refer to *MEN1* transcript NM_130799.2, protein NP_570711.1 (610‒amino acid isoform).

Abbreviation: HGVS, Human Genome Variation Society.

There is one major isoform (610 amino acids) and one minor isoform (615 amino acids) of menin in the sequence databases. The longer minor isoform could have originated with use of an alternative splice donor site in exon 1, such that the longer isoform contains five residues inserted at the end of exon 1 (at amino acid 148) that lead to a total 615‒amino acid coding region. Although the gnomAD and SGCD variants are annotated according to the 615-residue isoform encoded by transcripts NM_130803/ENST00000337652, the HGMD Pro and structural databases use the 610-residue isoform encoded by NM_130799/ENST00000312049 as the default. All numbering in this manuscript refers to the 610-residue form of menin, and variants from the gnomAD and SGCD have been reannotated accordingly.

### B. Protein Structures

Structures of human menin were downloaded as Protein Data Bank (PDB) files from the worldwide PDB [28]; a full list of the 29 crystal structures, containing 31 discrete menin chains, used in this analysis is shown in Table 2. Any nonnative amino acids (*e.g.,* affinity purification tags) in these structures were removed from PDB files before further analysis.

**Table 2.** MEN1 Crystal Structures Used in FoldX Analysis

PDB ID	Title	Resolution (Å)	Release Date	Menin Chain(s)	Used for RSA Analysis?	Reference
3u84	Crystal structure of human menin	2.50	15/2/2012	A, B	Yes (chain A)	29
3u85	Crystal structure of human menin in complex with MLL1 (KMT2A)	3.00	15/2/2012	A	Yes	
3u86	Crystal structure of human menin in complex with JunD	2.84	15/2/2012	A		
3u88	Crystal structure of human menin in complex with MLL1 (KMT2A) and LEDGF (PSIP)	3.00	15/2/2012	A, B	Yes (chain B)	
4gpq	Structural insights into inhibition of the bivalent menin-MLL interaction by small molecules in leukemia	1.46	19/9/2012	A		30
4gq3	Human menin with bound inhibitor MI-2	1.56	19/9/2012	A		
4gq4	Human menin with bound inhibitor MI-2-2	1.27	19/9/2012	A		
4gq6	Human menin in complex with MLL (KMT2A) peptide	1.55	19/9/2012	A		
4i80	Crystal structure of human menin in complex with a high-affinity macrocyclic peptidomimetic	3.10	6/3/2013	A	Yes	31
4og3	Human menin with bound inhibitor MIV-3R	2.01	5/3/2014	A		32
4og4	Human menin with bound inhibitor MIV-3S	1.45	5/3/2014	A		
4og5	Human menin with bound inhibitor MIV-5	1.63	5/3/2014	A		
4og6	Human menin with bound inhibitor MIV-4	1.49	5/3/2014	A		
4og7	Human menin with bound inhibitor MIV-7	2.08	5/3/2014	A		
4og8	Human menin with bound inhibitor MIV-6R	1.53	5/3/2014	A		
4x5y	Menin in complex with MI-503	1.59	15/4/2015	A		33
4x5z	Menin in complex with MI-136	1.86	15/4/2015	A		
5db0	Menin in complex with MI-352	1.50	30/3/2016	A		34
5db1	Menin in complex with MI-336	1.86	30/3/2016	A		
5db2	Menin in complex with MI-389	1.54	30/3/2016	A		
5db3	Menin in complex with MI-574	1.71	30/3/2016	A		
5dd9	Menin in complex with MI-326	1.62	9/9/2015	A		35
5dda	Menin in complex with MI-333	1.83	9/9/2015	A	Yes	
5ddb	Menin in complex with MI-319	1.54	9/9/2015	A		
5ddc	Menin in complex with MI-2-3	1.62	6/7/2016	A		
5ddd	Menin in complex with MI-836	2.14	9/9/2015	A		
5dde	Menin in complex with MI-859	1.78	9/9/2015	A		
5ddf	Menin in complex with MI-273	1.66	9/9/2015	A	Yes	
6b41	Menin bound to M-525	2.61	24/1/2018	A	Yes	36

A total of 29 PDB structures containing 31 menin chains were used for thermodynamic analysis using FoldX; seven representative structures were also used for relative solvent accessibility (RSA) analysis.

### C. *In Silico* Mutagenesis and Thermodynamic Analysis

Before *in silico* mutagenesis, sidechain repair and energy minimization were performed on all 31 menin chains in isolation, using the RepairPDB function of the FoldX modeling suite [2], version 4. The FoldX BuildModel function was then used to introduce individual substitutions into each of the repaired PDB structures. Of the 389 unique missense variants, 338 were covered by at least one PDB structure (pathogenic, n = 161; benign, n = 161; uncertain, n = 9; novel, n = 7). For each substitution, FoldX reported a change in free energy (*ΔΔG*) resulting from the substitution; from this, an average *ΔΔG* value was calculated for each variant across all structures containing the relevant position. In total, all 31 structures were used for 308 of 338 variants (mean for all variants = 29 structures), whereas analysis was possible using only a single structure for seven variants because of differences in coverage of individual PDB files. A full list of variants, the number of PDB structures analyzed for each, and average *ΔΔG* values for each variant have been published online [37]. All structures were visualized in PyMOL [38].

### D. Calculation of Solvent Accessibility

The absolute area accessible to solvent was calculated on a residue-by-residue basis for seven representative structures of menin using Define Secondary Structure of Proteins algorithm [39, 40] version 3.0.0 [41]. After an average area accessible to solvent value was calculated for each residue, relative solvent accessibility (RSA) was derived using the theoretical scale described by Tien *et al.* [42]. A list of structures used for DSSP analysis is included in Table 2.

## 2. Results

### A. Pathogenic Variants in MEN1 Are Predicted to Be More Destabilizing Than Benign Variants

**Figure 1.**
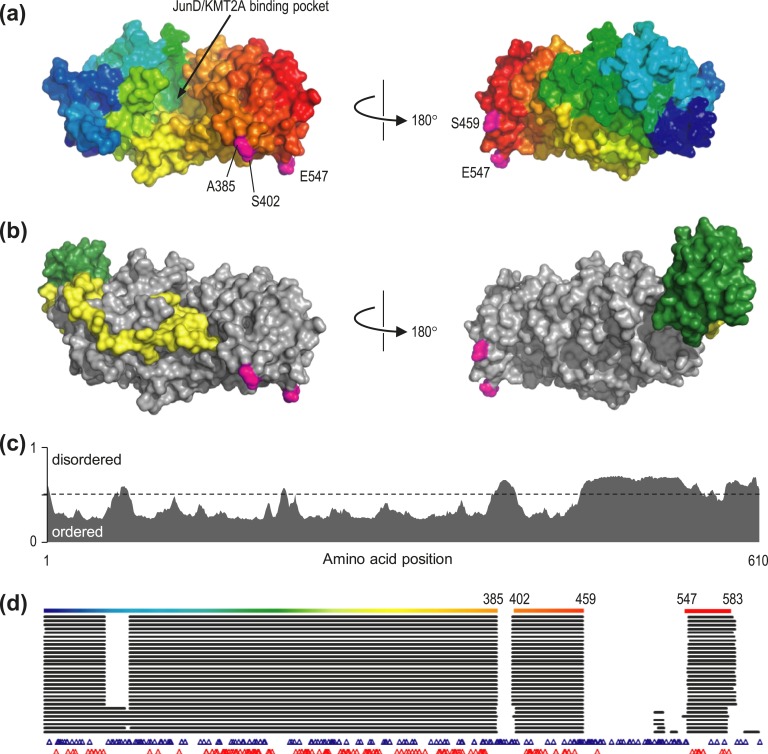
Structure and disorder in menin. (a) The structure of menin, as represented by PDB entry 3u88 chain A. The protein surface is colored from blue, *N*-terminal, to red, C-terminal; the position of the binding pocket for JunD and KMT2A is indicated (arrow). Magenta numbered residues indicate positions flanking disordered loops, which are not resolved in the crystal structure. (b) Menin (gray) in complex with KMT2A (yellow) and PSIP (green), as determined in PDB 3u88; note that although one end of KMT2A occupies the binding pocket, interaction with PSIP and other regions of KMT2A extends over a wider region of the menin surface. (c) Probability of intrinsic disorder in menin, as calculated by the MetaDisorder predictor, plotted against amino acid position. Extended regions of probability >0.5 are considered disordered. (d) Coverage of menin residues in the 31 PDB structures used in this analysis, aligned against amino acid position as in part (c). The top line shows coverage in PDB 3u88A, colored as in (a); numbering indicates residues flanking unstructured regions missing from the crystal structure. Below this, black horizontal lines show coverage for the 30 remaining PDB structures, whereas positions of benign and pathogenic variants are indicated by blue or red triangles, respectively. Note that regions of predicted intrinsic disorder are absent from the majority of, if not all, crystal structures, consistent with greater mobility of these residues within the crystal, and that few pathogenic variants have been reported in these regions.

More than 30 crystal structures were previously reported for menin [Fig. 1(a)]; most of these contain the protein in isolation or bound to a small (drug) ligand, whereas others show menin in complex with peptides from JunD, KMT2A, or PSIP [Fig. 1(b); Table 2]. Although all structures have been derived from expression of full-length (or near full‒length) menin, a number of regions remain unresolved in crystal structures. These regions predominantly lie in the C-terminal of the protein and correspond to stretches of predicted intrinsic disorder [43] in the protein [Fig. 1(c) and 1(d)], presumably resulting in high mobility within crystals. Interestingly, although these regions contain a similar distribution of benign variants as seen in the protein as a whole, inherited pathogenic variants are rare in regions of predicted disorder in menin [Fig. 1(d)]; however, we cannot rule out the possibility that the lack of pathogenic variants in these regions is due to reporting bias toward variants that lie close to those already known. Furthermore, recurrent missense variants have been observed in disordered regions of menin in the COSMIC database of somatic mutations in cancer [44], of which three (R479W, R485Q, and P540R) have not been reported in the gnomAD. It is therefore possible that missense variants in disordered regions play a role in pathogenicity, particularly when they arise as somatic mutations. With respect to inherited pathogenic variants, however, their distribution almost entirely within ordered regions means that the vast majority (161 of 162) are covered by one or more PDB entries and are thus amenable to structural analysis.

**Figure 2.**
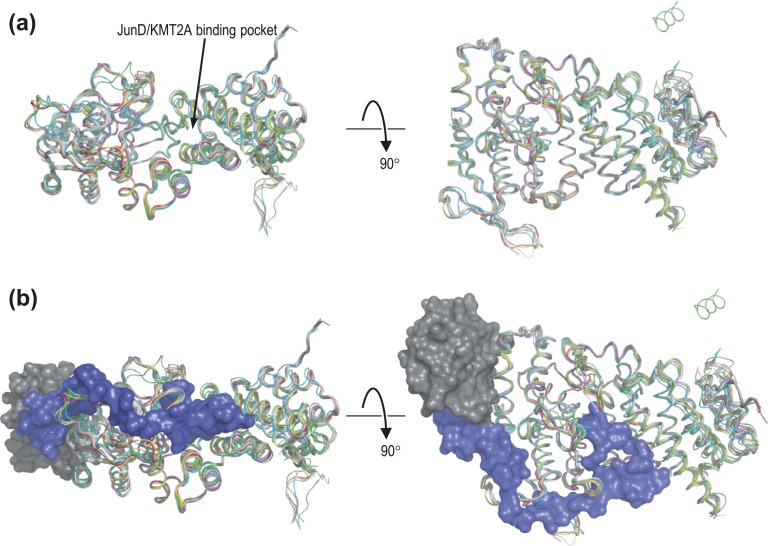
Alignment of menin structures. (a) The *α* carbon atoms of the 31 menin structures used in this study were aligned to that of PDB 6b41; each chain is shown in ribbon format, colored by PDB and chain identifier. The position of the JunD/KMT2A binding pocket is indicated. The short helix visible at the top right of the rotated figure corresponds to residues 596‒608 at the extreme C-terminal of menin, which were resolved only in PDB 3u84 chain A. (b) As in (a), but superimposed with the structures of MLL (blue) and PSIP (gray) from PDB 3u88.

The overall structure of menin is highly comparable to that within all reported PDB structures (alignment to PDB 6b41 yields an average root-mean-square deviation of 0.65 Å; range, 0.55 to 1.10 Å). Moreover, there is no significant effect of ligand binding on menin structure (Fig. 2). Because different PDB files contain slightly different numbers of amino acids but there are no obvious structural outliers, all available structures were used for thermodynamic analysis of missense variants *in silico* using FoldX.

**Figure 3.**
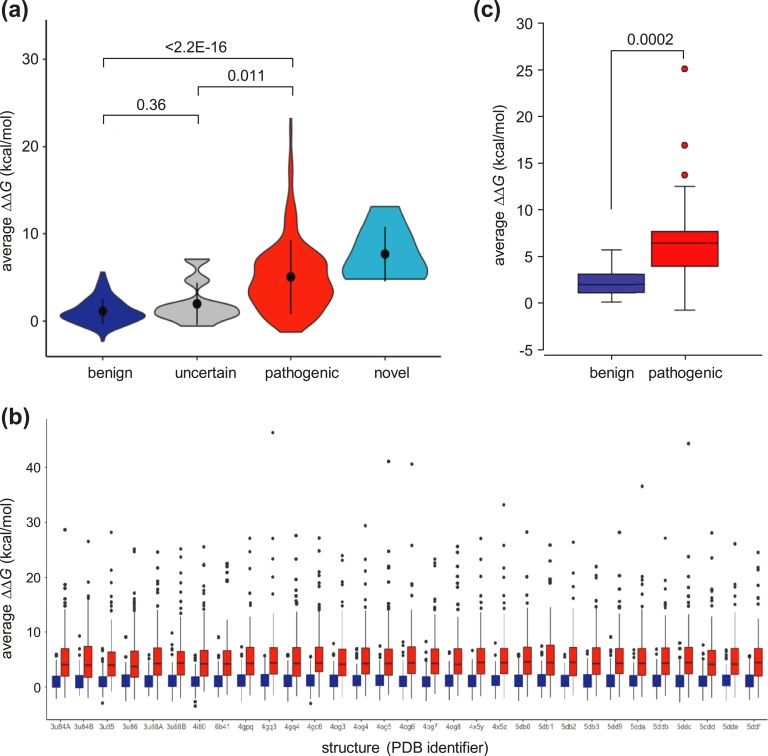
Pathogenic variants are predicted to destabilize menin structure. (a) *In silico* mutagenesis and thermodynamic analysis for menin variants. For each variant, the change in thermodynamic stability, *ΔΔG*, was calculated from all structures which contained the relevant residue and an average value calculated; average values for each variant were then plotted by variant group. Black circles and vertical lines within each data area represent median and upper and lower quartiles, respectively. Numbers above the data points show *P* values (Student two-tailed *t* test) between groups as indicated. (b) *ΔΔG* values for benign (blue) and pathogenic (red) variant groups were calculated for 31 individual PDB structures as shown on the *x*-axis. (c) Average *ΔΔG* values for benign and pathogenic variants occurring at the same amino acid position (residues with one benign and one pathogenic variant, n = 16; residues with two benign and one pathogenic variant, n = 5; residues with one benign and two pathogenic variants, n = 1). Colored boxes show the range between the upper and lower quartiles, and horizontal lines within each data box show median values; data points are shown for outliers only. The difference in the average *ΔΔG* value between groups was highly significant (*P* = 0.0002).

Benign and pathogenic variant groups were highly distinguishable by their predicted effect on thermodynamic stability, as represented by average *ΔΔG* value calculated across all structures. Variants resulting in *ΔΔG* >3 kcal/mol are generally regarded as strongly destabilizing [45]. The average *ΔΔG* value for all pathogenic variants was 5.06 kcal/mol (SD, 4.25 kcal/mol), with 108 of 161 of these (67.1%) predicted to be strongly destabilizing (*ΔΔG* > 3 kcal/mol). In contrast, the average *ΔΔG* value for putatively benign (gnomAD and SGCD) variants was 1.13 kcal/mol (SD, 1.46 kcal/mol), with only 17 of 161 (10.6%) having an effect in excess of 3 kcal/mol [Fig. 3(a)]. Of note, all seven novel missense variants were also predicted to be strongly destabilizing (average *ΔΔG*, 7.67 kcal/mol; SD, 3.14 kcal/mol; range, 4.81 to 13.16 kcal/mol). Analysis of *ΔΔG* values for individual PDB structures showed a similar separation of putative benign and pathogenic variant groups, with the vast majority of variants falling into a similar range for all structures [Fig. 3(b)].

We further compared the effect at multiallelic sites where different benign and pathogenic missense variants occur at the same position. Analysis of 27 benign and 23 pathogenic variants co-occurring at 22 residues again showed that the difference between the two groups was highly significant (*P* = 0.0002) and that, as a group, pathogenic missense changes were more strongly destabilizing than benign ones at the same position (average *ΔΔG* value by group = 6.81 and 2.18 kcal/mol, respectively) [Fig. 3(c)]. Therefore, because both pathogenic and benign variations can occur at the same site within menin, it is important to consider both the position and nature of the amino acid change; these data suggest that *ΔΔG* value may be useful in assessing the effect of novel variants at multiallelic sites.

**Figure 4.**
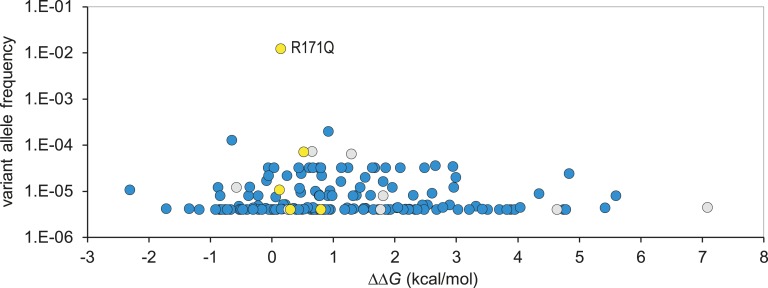
Population frequency of *MEN1* variants. The frequency of benign and uncertain missense variants in the gnomAD database were plotted against *ΔΔG* value. Blue fill: variants occurred in the gnomAD database only; yellow fill: variants reported in the gnomAD and SGCD databases; gray fill: variants reported in both gnomAD and HGMD Pro (DM class) databases. In cases where different nucleotide substitutions give rise to the same amino acid change, frequency is shown as a total for all variant alleles.

If variants that destabilize menin structure do indeed have a greater tendency to be pathogenic, it may be expected that the variants most frequently observed in the general population have the least destabilizing effect. This appears to be the case, as variants with the highest population frequency had average predicted *ΔΔG* values in the range of −1 to +1 (Fig. 4); because the error in FoldX calculations is approximately ±0.8 kcal/mol [2], this suggests little or no effect of these variants on protein stability. Of note, those variants—which have also been observed in a healthy aging population as represented by the SGCD cohort (median age, 80 to 85 years) and are therefore most likely to be truly benign—all occur within this range of *ΔΔG* values. This group includes the only commonly occurring missense *MEN1* variant, R171Q, which has an average *ΔΔG* value of 0.15 kcal/mol. Conversely, we noted that some variants reported in the gnomAD have *ΔΔG* values >4 kcal/mol; in fact, two of these nine variants (S38P and D315Y) have also been reported as disease causing in the HGMD Pro. As the symptoms of MEN1 often appear only in later life, it is not unexpected that some potentially pathogenic variants will be observed in the gnomAD database.

### B. Most Pathogenic Variants Are Buried in the Menin Structure

**Figure 5.**
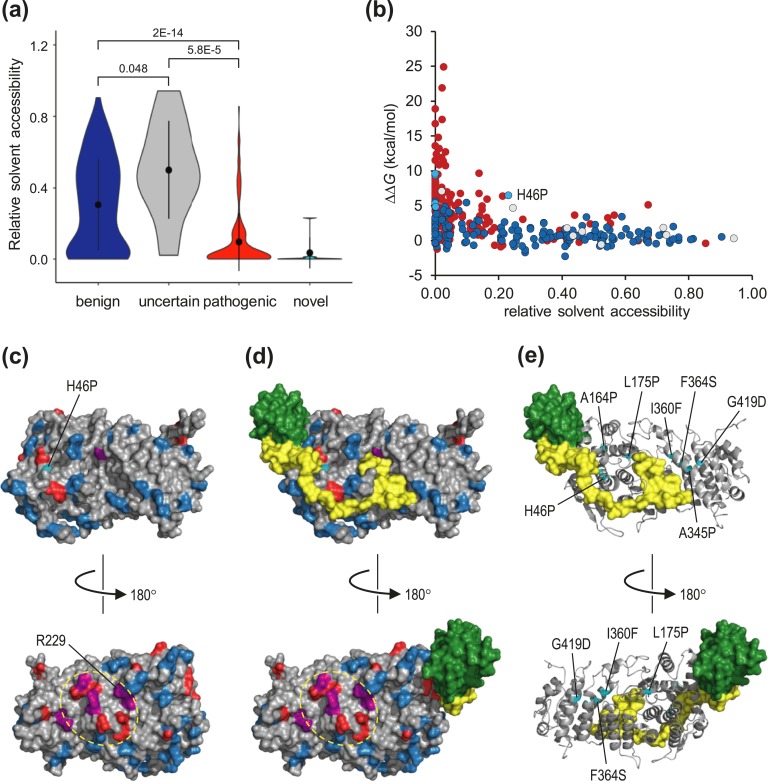
Molecular distribution of pathogenic and benign variants. (a) Relative solvent accessibility was calculated for each variant group. Black circles and vertical lines within each data area represent median and upper and lower quartiles, respectively. Numbers above data points show *P* values (Student two-tailed *t* test) between groups as indicated. (b) Buried pathogenic variants are predicted to be the most destabilizing to menin structure. Points indicate pathogenic (red), benign (dark blue fill), uncertain (gray fill) or novel (light blue fill) variants, respectively. Note that six of the seven novel missense variants reported here are deeply buried within the protein (RSA < 0.02), whereas only novel variant H46P is solvent accessible. (c and d) Surface distribution of solvent-accessible variants. The surface of menin (gray), either (c) alone or (d) in complex with KMT2A (yellow) and PSIP (green), shows all variants with RSA >0.2: blue, benign; red, pathogenic; purple coloring shows positions at which different pathogenic and benign variants were observed; the novel H46P variant is colored cyan. The broken yellow ovals indicate a cluster of pathogenic variants that may constitute an as-yet unidentified interface for protein-protein interactions. (e) Menin is shown as a gray ribbon; novel missense variants are colored cyan with sidechains displayed in stick format. KMT2A and PSIP are shown as in (d).

To examine whether there are differences in the spatial distribution of benign and pathogenic variants, we calculated the RSA of wild-type residues at all positions of missense substitutions [37]. This showed that although positions of benign variants are distributed throughout the volume of the protein, 86.3% of pathogenic variants occur in solvent-inaccessible (*i.e.,* buried) regions of RSA <0.2 [Fig. 5(a)]. Of note, this was also true for the seven novel variants, six of which had an RSA value <0.02. Plotting RSA against *ΔΔG* showed that variants at buried positions were also likely to be the most strongly destabilizing to protein structure [Fig. 5(b)]. Nevertheless, we observed that a significant number of pathogenic variants exhibited both accessibility to solvent (RSA > 0.2) and relatively low *ΔΔG*. Mapping the positions of solvent-accessible variants onto the surface of menin showed that, as for distribution throughout the internal volume of the protein, benign variants tended to be distributed across the surface. In contrast, pathogenic variants appeared to occur in clusters, one of which corresponded to binding surfaces for JunD, KMT2A, and PSIP [Fig. 5(c) and 5(d)], whereas another occurred on the menin surface opposite the JunD binding pocket. It is therefore possible that the latter region represents the site of an as-yet uncharacterized functional interaction of menin. As described previously, six of seven novel missense variants occurred at positions that were buried in the interior of the protein, whereas the only solvent-accessible variant, H46P, occurred at the interface with KMT2A and presumably acts to impair this interaction [Fig. 5(e)].

**Figure 6.**
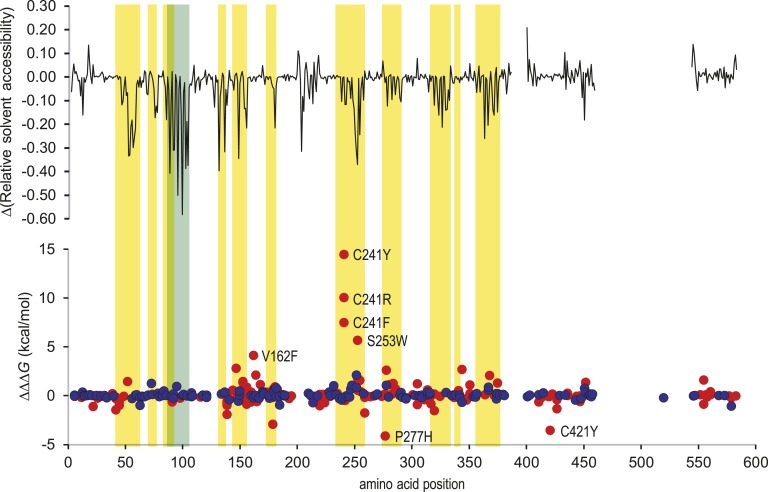
Effect of protein-protein interaction on *ΔΔG*. Analysis of solvent accessibility and thermodynamic effect of variants was performed on PDB 3u88 (menin:KMT2A:PSIP complex), both on menin chains in isolation (chains A, B) and as part of the complex. The upper graph shows the average difference in solvent accessibility by position in the complexed and isolated menin chains, respectively [*Δ*RSA = RSA (complex) − RSA (isolated)]. The lower graph shows the equivalent difference in average *ΔΔG* value at each position (*i.e., ΔΔΔG*). Data points are labeled for variants for which *ΔΔΔG* 3 kcal/mol (red, pathogenic; dark blue, benign). Background shading indicates positions of menin residues forming contacts with KMT2A (yellow) or PSIP (green) in PDB 3u88.

To further investigate the effects of protein interactions on the thermodynamic effects of *MEN1* variants, we compared *ΔΔG* values for variants in PDB structure 3u88 (menin complexed with KMT2A and PSIP peptides) by analysis of both menin chains in isolation (chains A, B) and those complexed to KMT2A and PSIP. As expected, regions of decreased solvent accessibility in the complexes aligned with residues annotated as forming protein-protein contacts (Fig. 6). However, the presence of bound peptides had little effect on *ΔΔG* values of benign variants, indicating that these have a neutral effect on protein binding. Conversely, protein binding had a large effect on *ΔΔG* values of a number of pathogenic variants; again, these predominantly occurred at or close to protein interfaces, indicating that these variants are likely to have a direct effect on ligand binding by menin.

### C. Destabilizing Variants Reduce Levels of Functional Menin Protein

**Figure 7.**
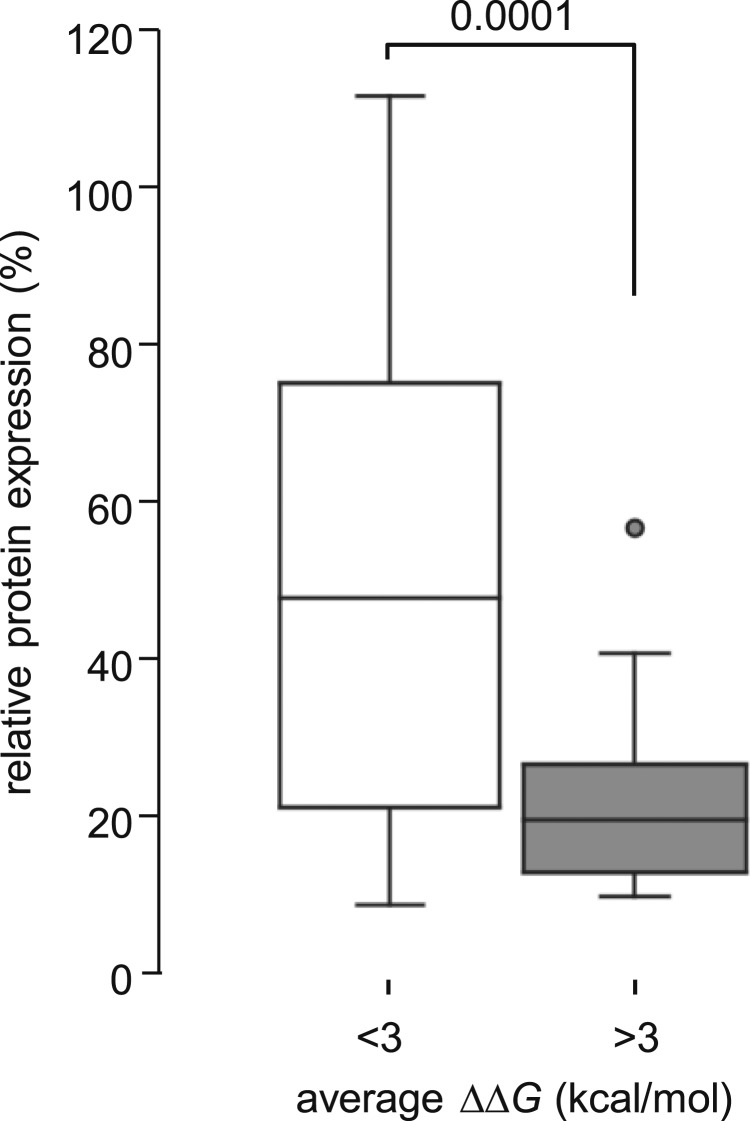
Predicted thermodynamic stability correlated with observed expression. (a) Steady-state expression levels have been reported for a number of menin variants; relative expression level data were sorted into two groups according to *ΔΔG* value as calculated in this study [neutral or weakly destabilizing: *ΔΔG* < 3 kcal/mol (n = 14); strongly destabilizing: >3 kcal/mol (n = 27)]. Boxes show the range between upper and lower quartiles; horizontal lines within each data box show median value. Data points are shown for outliers only. The difference in relative expression between the two groups was highly significant (*P* = 0.0001).

Previous reports on the effects of missense variants on levels of functional menin within the cell showed that pathogenic variants have a tendency to increase protein turnover and/or reduce the steady-state level of protein, whereas benign variants tend to have no such effect [46, 47]. We correlated the previously reported effects of variants on levels of steady-state protein with average *ΔΔG* values and observed that variants that were predicted to be strongly destabilizing *in silico* (*ΔΔG* > 3 kcal/mol) exhibited significantly lower levels of steady-state protein in cell-based assays (*P* = 0.0001) (Fig. 7), consistent with the hypothesis that variants with high *ΔΔG* values reduce the biological activity of menin.

### D*.* Can *ΔΔG* Value Be Used as an Aid to Variant Classification?

**Figure 8.**
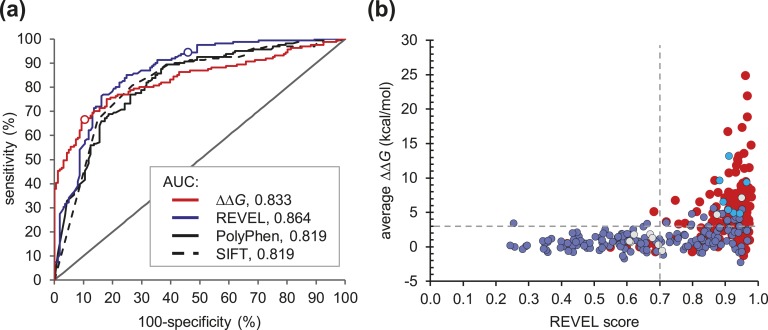
Use of thermodynamic analysis to assess the effect of novel missense variants. (a) Receiver operating characteristic curves for groups of pathogenic and benign variants as functions of *ΔΔG* value [red line; area under the curve (AUC), 0.833], REVEL score (blue line; AUC, 0.864), PolyPhen2 probability for pathogenicity (black line; AUC, 0.819), and SIFT score (broken black line; AUC, 0.819). Open circles on *ΔΔG* and REVEL traces indicate positions corresponding to threshold values of 3 and 0.7 kcal/mol, respectively. (b) Scatter plot of *ΔΔG* value against REVEL score for all variants (red circles, pathogenic; blue fill, benign; gray fill, uncertain; cyan fill, novel). Where different nucleotide substitutions gave rise to the same amino acid change, the REVEL score was calculated as an average of values for the individual nucleotide variants. Broken horizontal and vertical lines indicate thresholds of *ΔΔG* = 3 kcal/mol and REVEL score = 0.7, respectively. Note that all seven novel missense variants are clustered in the upper right quadrant of the plot.

To evaluate the clinical validity of *ΔΔG* values, we performed receiver operating characteristic curve analysis for the groups of benign and pathogenic variants and compared the results with the outputs from a number of commonly used phenotypic predictions tools: SIFT [48], PolyPhen [49], and REVEL [50]. All methods yielded areas under the curve of 0.819 to 0.864, indicating that all have clinical validity [Fig. 8(a)]. However *ΔΔG* analysis resulted in the highest specificity but lowest sensitivity. *ΔΔG* values >3 kcal/mol are generally regarded as being strongly destabilizing toward protein structure [45]. Taking this as a threshold for variant classification gave a sensitivity and a specificity of 67.1% and 89.4%, respectively (PPV, 86.4%), whereas setting a more conservative threshold of ≥4 kcal/mol increased the specificity to 95.0%, though with a concomitant loss of sensitivity (54.0%; PPV, 90.6%). A marginal increase in PPV could be obtained by combining *ΔΔG* thresholds with a cutoff in the REVEL score of 0.7, which has been reported to exclude 95% of false-positive calls [51], yielding PPVs of 87.7% at *ΔΔG* ≥3 kcal/mol and 91.5% at *ΔΔG* ≥4 kcal/mol. Of note, all seven novel missense variants reported here cluster within the upper right quadrant [Fig. 8(b)], consistent with a severe effect on protein stability and suggesting that *ΔΔG* values can potentially be used to provide evidence toward variant classification in MEN1.

## 3. Discussion

Previous work has shown that predicted thermodynamic destabilization of protein structure, as measured by *ΔΔG* values calculated by FoldX, can be used as a predictor of pathogenicity in *MSH2* and *PAH* variants [7, 8]. Our data indicate that the same is true for variants in *MEN1* and that a high predicted *ΔΔG* value is a strong positive predictor for pathogenicity. A threshold value of *ΔΔG* >3 kcal/mol, which is generally regarded as strongly destabilizing, yielded a specificity of 89.4% for classification of menin variants, rising to 95.0% for a more conservative threshold of 4 kcal/mol. By contrast, using a proposed threshold of 0.7 for the phenotypic meta-prediction tool REVEL yielded a specificity of only 53%. Although MEN1 has a high degree of penetrance, with more than 95% of individuals with pathogenic variants expected to develop symptoms by the sixth decade of life [9], there appears to be no correlation between MEN1 variants and clinical manifestations, with interfamilial and intrafamilial variations observed [22]. Therefore, the identification of likely pathogenic variants has important implications for patient surveillance and genetic testing of family members. For example, analysis of a large cohort of Florentine patients showed that age at genetic diagnosis in relatives of the index cases was 31.2 + 16.9 years, with a range of 1 to 71 years [21]. With respect to the seven novel missense variants reported here, all had high average predicted *ΔΔG* values (range, 4.81 to 13.16 kcal/mol), and six were deeply buried within the protein, strongly supporting pathogenicity. All these cases were also predicted as deleterious or probably pathogenic by commonly used tools for *in silico* pathogenicity prediction; however, the comparatively low specificity of all these tools for variants in *MEN1* highlights the value of thermodynamic analysis as a means of reducing false-positive calls.

As might be expected, our analysis showed that variants that are buried within the menin structure are predicted to result in greatest structural destabilization. In fact, the majority (86.3%) of reported germline pathogenic variants in *MEN1* are buried, suggesting that any novel variant that is solvent inaccessible (RSA < 0.2) and has a predicted *ΔΔG* >4 kcal/mol is also highly likely to be pathogenic. Nevertheless, a number of pathogenic variants lie on the surface of menin, and many of these have relatively low *ΔΔG* values. A number of these variants lie at or close to positions of known interactions with binding partners, such as JunD, KMT2A, or PSIP, where they presumably have an adverse effect on binding of these factors, emphasizing the value of integrating all known structural annotations into a final classification of the likely effect of a variant.

Our data also suggest the possible existence of an as-yet unidentified interaction of menin, as evidenced by the cluster of pathogenic variants lying on the protein surface opposite the JunD binding pocket. Interestingly, in a recent analysis of the spatial distribution of missense variants [52], *MEN1* was identified as one of a group of genes displaying substantial spatial clustering of pathogenic and likely pathogenic missense variants in the ClinVar database [53], but not of benign or likely benign missense variants reported in the gnomAD. Inspection of ClinVar (accessed 19 September 2019) revealed that of 346 missense variants in *MEN1*, only 50 unique variants (excluding start-loss variants) were classified as pathogenic or likely pathogenic, with a large majority (276) classed as being of uncertain significance. Although we used a different database, HGMD Pro, as the source for our data set of “pathogenic” variants, there is considerable overlap between the two, with 39 of 50 (78%) of the pathogenic or likely pathogenic ClinVar variants also being present in our data set, whereas a further 27 variants in our data set were colocalized with a ClinVar variant. However, the discrepancy between the total number of pathogenic variants in the two data sets highlights the need for more reliable tools for classification of variants in *MEN1*.

In terms of the broader applicability of this approach, our work builds upon the reported analysis of *MSH2* and *PAH* variants and applies it to the classification of novel clinical variants. Whether the same approach can be used for other proteins remains to be determined. One obvious limitation of structural analysis is, by definition, the need for a suitable structural model. However, even when no experimental structures are available for a protein of interest, it may still be possible to use comparative modeling to generate a reliable model of regions or domains for structural analysis. Another likely limitation is the architecture of the protein itself. Both menin and MSH2 are relatively compact, globular proteins, with low surface area/volume ratio and a high proportion of amino acids in regions of secondary structure. As a result, the effect of missense variants on the internal geometry and thermodynamic stability of the proteins is amenable to *in silico* prediction, particularly given the availability of suitable high-quality PDB structures. It seems likely, therefore, that the approach used here has broader applicability in proteins that contain a high proportion of buried residues in regions of strong secondary structure. Indeed, the potential for wider use of *in silico* thermodynamic analysis of protein stability as part of a pipeline for assessing the effect of missense variants was recently reviewed [54]. However, less well-structured proteins, or fibrillar proteins in which a greater proportion of amino acids are exposed to solvent, are likely to be less amenable to such study, as the confidence with which the structural and thermodynamic effects of missense variants can be predicted will be greatly reduced. Such rules are likely to be revealed only by a proteome-wide study, which is beyond the scope of this manuscript.

In summary, we have shown that structural analysis of missense substitutions in *MEN1* can be used to identify variants that are likely to destabilize the protein and thus potentially aid in variant classification. Given that all analyses described herein used publicly available data and freely available software that did not require specialist bioinformatic skills or infrastructure, such analysis lies within the capability of any genetics laboratory or testing service. As such, there is significant scope for making greater use of protein structural data in the routine interpretation of genetic variations.

## Abbreviations:

DM: disease mutation
gnomAD: Genome Aggregation Database
HGMD Pro: Human Gene Mutation Database, Professional version
MEN1: multiple endocrine neoplasia type 1
PAH: phenylalanine hydroxylase
PDB: Protein Data Bank
PPV: positive predictive value
RSA: relative solvent accessibility
SGCD: Sydney Genomics Collaborative Database
*Δ**Δ**G*: change in free energy
